# Quality by Design Based Chromatography Technique Development and Validation for the Medicine Venetoclax (for Chronic Leukemia), in the Context of Impurities Including Degradation Products

**DOI:** 10.1002/bmc.70072

**Published:** 2025-04-02

**Authors:** Rajeshwari Dandabattina, Karuna Sree Merugu, Lova Gani Raju Bandaru, Haridasyam Sharathbabu, Rambabu Gundla, Naresh Kumar Katari

**Affiliations:** ^1^ Bhavans Vivekananda College of Science, Humanities and Commerce Secunderabad Telangana India; ^2^ Department of Chemistry, GITAM School of Science GITAM Deemed to be University Bengaluru Karnataka India; ^3^ Department of Chemistry, GITAM School of Science GITAM Deemed to be University Hyderabad Telangana India; ^4^ School of Chemistry & Physics, College of Agriculture, Engineering & Science, Westville Campus University of KwaZulu‐Natal Durban South Africa; ^5^ R&D, CHEMTEX Environmental Laboratories Inc. Port Arthur Texas USA

**Keywords:** experimental design, method development, quality by design, validation, venetoclax

## Abstract

The present research study describes the Venetoclax (VEN)‐related substances test method using RP‐HPLC/DAD techniques. It was developed and validated according to ICH Q14 and Q2(R2) guidelines. The substances were separated using an X‐Bridge Phenyl column (150 mm × 4.6 mm, 3.5 μm) and a gradient program. The mobile phase A, consist 0.02 mM Na2HPO4 (pH 8.0) buffer and acetonitrile in an 80:20 v/v ratio. Mobile phase B was prepared using a 75:25 v/v mixture of acetonitrile and a pH 8.0 buffer and well mixed. The flow rate remains constant at 1.0 mL/min, traversing an appropriate gradient program. The VEN and its impurities were detected at 280 nm, with an injection volume of 15 μL and a runtime of 130 min. Moreover, we identified proper degradation impurities and sensitivity of VEN due to forced‐degradation study experiments. The linearity and range of the testing procedure were validated by computing *r* ^2^ values over 0.999. All organic impurities were recovered at a rate of 97.6%–106.0% with a relative standard deviation of 0.11%–4.35%. A robustness test was conducted utilizing the AQbD methodology. The proposed method was stability‐indicating in nature and can be used for commercial samples in the pharmaceutical industries.

## 1 Introduction

**FIGURE 1:**
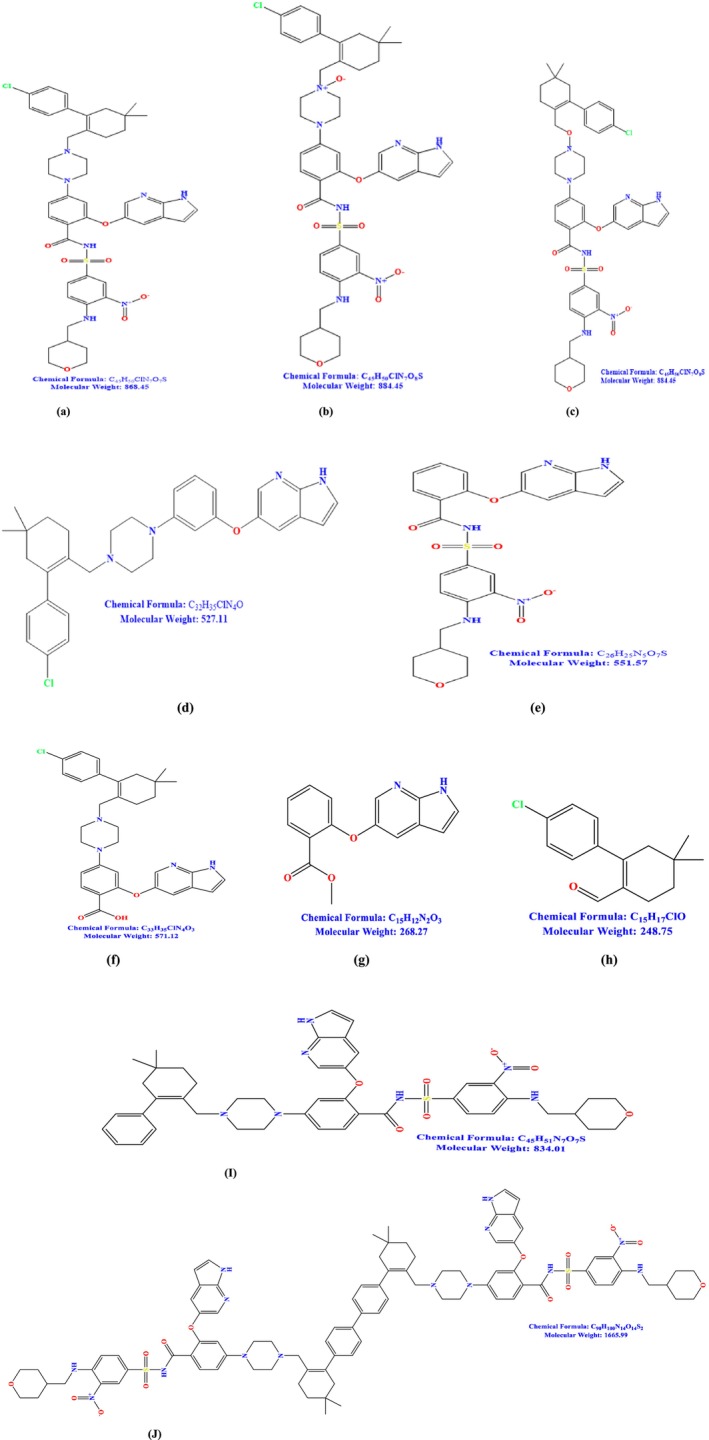
(a) Venetoclax, (b) Venetoclax‐N oxide (Impurity‐II), (c) Venetoclax oxidative impurity, (d) Venetoclax (Impurity‐III), (e) Impurity G, (f) Impurity D, (g) Impurity E, (h) Impurity C, (i) Impurity A, (j) Impurity B.

Venetoclax (VEN) is a specific inhibitor of the anti‐apoptotic protein B‐cell Lymphoma 2. It is utilized for the treatment of chronic lymphocytic leukemia with 17p deletion (Vaxman et al. 2018; Leverson et al. 2017). VEN is around tenfold more effective than navitoclax in inducing apoptosis in chronic lymphocytic leukemia (CLL) (Juárez‐Salcedo et al. 2019; Mihalyova et al. 2018). It is a solid ranging from pale yellow to dark yellow, with the empirical formula C_45_H_50_ClN_7_O_7_S and a molecular weight of 868.44. VEN is chemically characterized as 4‐(4‐{[2‐(4‐chlorophenyl)‐4,4‐dimethylcyclohex‐1‐en‐1‐yl]methyl}piperazin‐1‐yl)‐N‐({3‐nitro‐4‐[(tetrahydro‐2H‐pyran‐4‐ylmethyl)amino]phenyl}sulfonyl)‐2‐(1H‐pyrrolo[2,3‐b]pyridin‐5‐yloxy)benzamide, accompanied by the chemical structures of the active pharmaceutical ingredient (API) and its associated impurities depicted in the Figure 1. VENCLEXTA tablets for oral use are available as pale yellow or beige tablets containing 10, 50, or 100 mg of VEN as the active component. VEN is classified as a BCS class IV compound (Riedmaier et al. 2018), exhibiting low water solubility ( < 0.0042 μg/mL at pH 7.4) and significant hydrophobicity (Log *p* = 8.1). VEN has seven process impurities and two degrading impurities.

A literature study revealed that many chromatographic techniques have been employed for the quantification of VEN in pharmaceutical formulations (Zigart and Casar 2020). Although many studies assessed VEN in conjunction with other medication combinations, only a limited number exclusively evaluated VEN and its degradation products ( David Raju 2022). VEN estimation and metabolite assessment in plasma samples have been conducted mostly by LC–MS and HPLC methodologies (Yasu et al. 2022; Eisenmann et al. 2020; Fukuda et al. 2022; Divya Bhargavi et al. 2023). Nonetheless, most of these techniques encounter issues with mobile phase buffers, columns, and mobile phase pH. Furthermore, the resolution between impurities is insufficient, and forced degradation was not conducted in accordance with the International Conference on Harmonisation guidelines (ICH Harmonised Tripartite Guideline 2003; ICH Harmonised Tripartite Guideline 1996; ICH Harmonised Guideline 2023). Moreover, the documented methodologies did not encompass all VEN impurities, including process‐related and degrading impurities.

The aim of this work was to provide a straightforward, specific, linear, robust, tough, and stability‐indicating technique for quantifying VEN and its associated impurities in pharmaceutical formulations. The stability‐indicating technique is a proven quantitative analytical approach that often includes forced deterioration and validation experiments (Bakshi and Singh 2002). Building on these prior findings, we conducted degradation experiments and identified two possible impurities in the medicinal product during stress testing in the presence of peroxides. The chemical exhibits stability under both physical and hydrolytic degradation conditions, as corroborated by validation criteria. This approach is successful for identifying VEN and its associated impurities while being cost‐efficient.

The existing regulatory guidelines explicitly mandate that the methodology must be refined or demonstrated to be resilient via the statistical technique Quality by Design (QbD) (Bandaru et al. 2024; Rathnakar et al. 2023). The efficacy of the presently offered technique was shown by the application of the QbD tool. The Design of Experiments (DoE) utilizing QbD was conducted using Design Expert software version 13.

## 2 Methods and Materials

### 2.1 Instrumentation and Software

The experiment was conducted utilizing a Water Alliance 2695 HPLC (Water Corporation, Milford, MA, USA), which included a quaternary pump, a column thermostat, and an auto sampler, along with a Waters 2489 PDA detector. Instrument control and data capture were executed via Waters Empower 3 software. A pH meter (Eutech) was employed to ascertain the pH of the buffer. An ultrasonication device, analytical balances, and a vacuum microfiltration machine equipped with 0.22 μm PVDF filters from Millipore were utilized.

### 2.2 Chemicals, Reagents, and Reference Materials

AR grade disodium hydrogen phosphate was acquired from SD Fine Chemical, Mumbai, India; HPLC grade acetonitrile was obtained from Merck, Mumbai, India; and Emparta ACS grade orthophosphoric acid was sourced from Merck, Mumbai, India. Milli‐Q water was procured from Millipore. Tablets procured from a local pharmacy in Hyderabad, India. The working standard of VEN and associated impurities were supplied by the SVAK Life Sciences Hyderabad, India.

### 2.3 Chromatographic Parameters

The buffer was prepared using 0.02 M disodium hydrogen phosphate, and the pH was adjusted to 8.0 using 5% orthophosphoric acid, thereafter filtered through a 0.22 μm membrane filter. Mobile phase A consisted of buffer and acetonitrile in an 80:20 v/v ratio, whereas mobile phase B included buffer and acetonitrile in a 25:75 v/v ratio. The impurities were isolated by X‐Bridge Phenyl (150 mm × 4.6 mm, 3.5 μm). Gradient elution was conducted at a flow rate of 1.0 mL min. The column and sample temperatures were sustained at 30°C and 5°C, respectively. The analytes and impurities were monitored at 280 nm in ultraviolet light. The injection volume is 15 μL, and the entire duration of the run is 130 min, utilizing a gradient program (Table 1). The washing solution employed consisted of water, acetonitrile, and orthophosphoric acid at a volumetric ratio of 500:500:1.

**TABLE 1:** Optimized gradient program.

Time (minutes)	%Mobile phase A	%Mobile phase B
0.01	82	18
5	82	18
10	68	32
40	55	45
45	55	45
60	45	55
85	30	70
95	25	75
100	5	95
118	5	95
120	82	18
130	82	18

### 2.4 Diluent Preparation

The diluent was prepared at a ratio of 0.1% OPA and acetonitrile at 48:52 v/v, respectively.

### 2.5 Analytical Solutions

#### 2.5.1 Preparation of the VEN Standard Solution

A VEN standard solution was prepared with a concentration of about 2 μg/mL by precisely weighing 20.2 mg of the VEN working standard, depositing it into a 100 mL volumetric flask, adding approximately 70 mL of diluent, sonicating to achieve dissolution, and then diluting to the final amount with diluent (Stock solution). Further transfer 2 mL of the above stock solution was put into a 200 mL volumetric flask containing diluent and well mixed.

#### 2.5.2 Sample Preparation Procedures

A sample solution of VEN was prepared with a concentration of about 1000 μg/mL by weighing a minimum of 20 tablets and calculating the average weight. Grind the pills into a fine, uniform powder. Weigh and transfer the tablet powder corresponding to 50 mg of VEN into a 50 mL volumetric flask. Introduce about 30 mL of diluent and sonicate for 20 min with periodic agitation, then adjust to the desired amount with diluent and ensure thorough mixing. Centrifuge the solution at 5000 RPM for 10 min and utilize the supernatant.

#### 2.5.3 Preparation of the Placebo Solution

Measured and put the placebo corresponding to about 50 mg of VEN into a 50 mL volumetric flask. Introduce about 30 mL of diluent and sonicate for 20 min with periodic agitation, then adjust to the desired amount with diluent and ensure thorough mixing. Centrifuge the solution at 5000 rpm for 10 min and utilize the supernatant.

#### 2.5.4 Preparation of Spiked Samples in Accordance With Specifications

Impurities were precisely prepared at concentrations of approximately 2 μg mL^‐1^ by weighing and transferring 2.5 mg of each process‐related impurity (Impurity A, Impurity B, Impurity C, Impurity G, Impurity K, Impurity D), and degradant impurities (Oxidative VEN, VEN Impurity – II, and VEN Impurity – III) into a 100 mL volumetric flask to prepare the Impurity Stock solution. We introduced 5 mL of impurity stock solution into a 50 mL volumetric flask holding the sample solution.

## 3 Results and Discussion

### 3.1 Methodology Development

#### 3.1.1 Enhancement of Chromatographic Parameters

**FIGURE 2:**
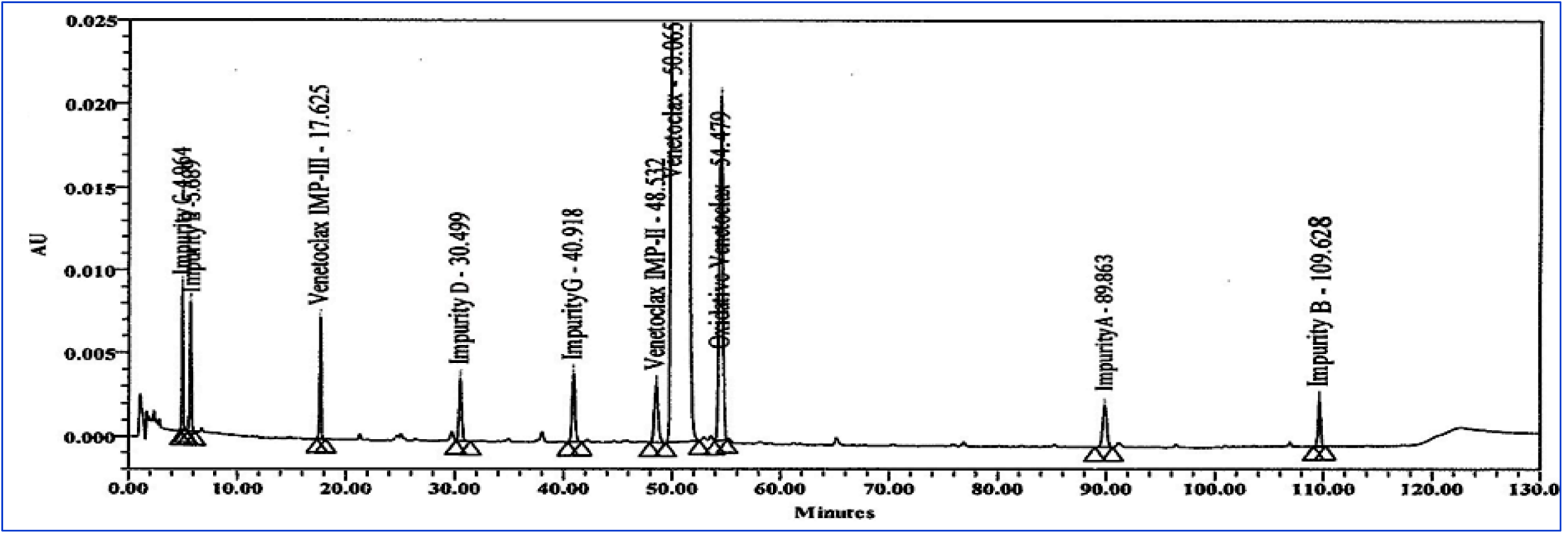
Venetoclax finished product spiked sample chromatogram.

This research work is really significant. It was produced by scientific and logical methods with appropriate technological rationale. Initially, we collected critical information like physicochemical qualities, pharmacopeial testing methodologies, research publications, stability data, and molecule‐specific details pertaining to VEN API and the completed product. The literature indicates that the VEN molecule has low polarity. Consequently, we originally selected an X bridge C8 column (150 mm × 4.6 mm, 5 μm) and an ammonium buffer at pH 4.0. Subsequently, blank, placebo, and spiked sample solutions were prepared in accordance with the specified specifications. Moreover, they were accurately administered into the LC‐DAD apparatus. The data indicate that it was not utilized. Subsequently, we conducted one further experiment; nevertheless, it proved to be useless. Additional information is available in Table 2. Various stationary phases were employed in the subsequent testing, including X‐Bridge Phenyl (150 mm × 4.6 mm, 3.5 μm). Phenyl stationary phases engage with molecules including aromatic groups or unsaturated bonds via π‐π interactions. A 20 mM solution of disodium hydrogen phosphate buffer was used to enhance the separation of impurities. Subsequently, we experimented with isocratic and gradient protocols in many configurations. Consult (Table 2 and Figure 2) for enhanced conclusions. Following the optimization of the testing procedure, we assessed feasibility metrics including specificity, accuracy, precision, linearity, solution stability, and performed forced‐degradation experiments. The findings indicate that the suggested testing procedure would successfully meet the validation criteria outlined in ICH Q2(R2) and USP 1225 recommendations.

**TABLE 2:** Method development and optimization trials.

No. of experiments	Method details	Column	Results and observations
Experiment 1	Mobile phase: 20 mM ammonium acetate buffer, pH 4.0: acetonitrile (60:40 v/v), flow rate: 1.0 mL/min, injection volume: 10 μL, UV: 280 nm, runtime: 60 min. (Isocratic mode).	X bridge C8 column (150 mm × 4.6 mm, 5 μm), temp: 25°C	Poor resolution between Impurity – II and the main peak. Peak purity test failed both. This trail was no use.
Experiment 2	Mobile phase: 20 mM Disodium hydrogen phosphate (pH 8.0): acetonitrile (70:30 v/v), flow rate: 1.0 mL/min, injection volume: 10 μL, UV: 280 nm, runtime: 80 min. (Isocratic mode).	X bridge C8 column (150 mm × 4.6 mm, 5 μm), temp: 30°C	Poor resolution between Impurity – II and the main peak. Impurity D and Impurity G both are merged. Peak purity test failed Impurity D and Impurity G. This trail was no use.
Experiment 3	Mobile phase: 20 mM Disodium hydrogen phosphate (pH 8.0): acetonitrile (70:30 v/v), flow rate: 1.0 mL/min, injection volume: 10 μL, UV: 280 nm, runtime: 110 min. (Isocratic mode).	X‐Bridge Phenyl (150 mm × 4.6 mm, 3.5 μm), temp: 30°C	The resolution was 1.3 obtained between Impurity – II and the main peak. The resolution was 1.1 obtained between Impurity D and Impurity G. All impurities responses are low. From this column, better performance was observed compared with previous performance. This trail was no use.
Experiment 4	Mobile phase A was composed of buffer and acetonitrile in a ratio of 80:20 v/v, and mobile phase B was composed of buffer and acetonitrile in a ratio of 25:75 v/v. Flow rate: 1.0 mL/min, injection volume: 15 μL, UV: 280 nm, runtime: 110 min. (Gradient mode).	X‐Bridge Phenyl (150 mm × 4.6 mm, 3.5 μm), temp: 30°C; sample cooler temp: 5°C	The resolution was 1.8 between Impurity – II and the main peak. The resolution was 2.5 obtained between Impurity D and Impurity G. All impurities responses are satisfactory. Better performance was observed in this trial compared with previous performance.
Experiment 5	Mobile phase A was composed of buffer and acetonitrile in a ratio of 80:20 v/v, and mobile phase B was composed of buffer and acetonitrile in a ratio of 25:75 v/v. Flow rate: 1.0 mL/min, injection volume: 15 μL, UV: 280 nm, runtime: 130 min. (Gradient mode).	X‐Bridge Phenyl (150 mm × 4.6 mm, 3.5 μm), temp: 30°C; sample cooler temp: 5°C	The resolution was 2.1 between Impurity – II and the main peak. A resolution of 3.5 was obtained between Impurity D and Impurity G. All impurities and degradation impurities are well separated in the trial. All impurities responses are satisfactory (Figure 2). Good performance was observed in this trial compared with previous performance. Based on the results, this method must be validated according to regulatory guidelines.

#### 3.1.2 Method Validation Study

This is an innovative, novel approach for identifying VEN‐related compounds in completed goods, with no compendial test method offered. A variety of performance characteristics were assessed, including system appropriateness, specificity, sensitivity, accuracy, precision, linearity, range, limit of detection (LOD), limit of quantification (LOQ), robustness, and solution stability, in accordance with ICH Q2(R2) and USP<1225>criteria (United States Pharmacopeial 2020; U.S. FDA 2015).

#### 3.1.3 System Suitability

The system's appropriateness was assessed using six duplicate injections of a standard solution and one injection of the LOQ solution, in accordance with the guidelines provided by the United States Pharmacopeia (USP). The peak asymmetry, theoretical plates, and percentage relative standard deviation (%RSD) for the primary peak regions were computed. The findings of the system appropriateness are shown in Table 5.

#### 3.1.4 Specificity and Forced‐Degradation Studies (Zigart and Casar 2020; Bandaru et al. 2023)

The method's specificity was assessed by injecting a blank placebo, individual impurities at a concentration of 2 μg mL^−1^, a standard solution, and a spiked sample solution. The approach demonstrated specificity, as no interference was detected in the blank and placebo chromatograms during the retention times (RTs) of the primary peak and impurities. The individual RTs, relative retention times (RRTs), purity angle, and purity threshold are presented in Table 3. The method's specificity is assessed by forced‐degradation trials in accordance with ICH Q1A criteria. The sample degradation was conducted under the following experimental conditions.

**TABLE 3:** Specificity and peak purity summary.

Peak name	Retention time (minutes)					
	Standard solution	Individual standard solution	Spiked sample	Relative retention time	Purity angle	Purity threshold
VEN	52.23	NA	50.07	1.00	0.150	0.427
VEN Impurity – III	NA	17.59	17.63	0.35	0.082	0.366
VEN Impurity – II	NA	48.68	48.54	0.97	0.229	0.519
*Oxidative VEN	NA	54.52	54.48	1.09	0.031	0.233
*Impurity C	NA	4.90	4.96	0.10	0.118	0.311
*Impurity E	NA	5.69	5.69	0.11	0.065	0.308
*Impurity D	NA	30.54	30.50	0.61	0.126	0.572
*Impurity G	NA	40.97	40.92	0.82	0.101	0.482
*Impurity A	NA	89.82	89.86	1.80	0.163	0.651
*Impurity B	NA	109.90	109.63	2.19	0.134	0.603

* Process impurities for information purposes only.

#### 3.1.5 Acid Degradation Study

Measured and transferred about 50 mg of the sample into a 50 mL volumetric flask, added 5 mL of 5 N HCl solution, and maintained the sample in a water bath at 80°C for 6 h. The sample was let to cool to room temperature and subsequently neutralized with 5 mL of 5 N NaOH solution. Incorporated 30 mL of diluent and subjected the mixture to sonication for 10 min to achieve dissolution. The volume was adjusted to the mark using diluent and well mixed. The sample underwent centrifugation at 5000 rpm for 10 min, and the resultant supernatant was utilized for HPLC analysis. The resulting chromatogram indicates no substantial deterioration in acidic conditions. The outcomes of the percentage test, percentage degradation, mass balance, and peak purity of VEN are presented in Table 4.

**TABLE 4:** Forced‐degradation conditions and data.

Stress condition	%Assay	%Degradation	Total related substance (%w/w)	%Mass balance	Purity angle	Purity threshold
As such	98.52	NA	0.70	NA	0.028	0.222
Acid degradation (5 N HCl at 80°C for 6 h	97.22	1.30	1.29	98.51	0.028	0.225
Base degradation (5 N NaOH at 80°C for 3 h	98.01	0.51	0.53	98.54	0.027	0.226
Thermal degradation (120°C for 24 h)	94.54	3.98	3.18	97.72	0.028	0.239
Photolytic degradation (1.2 million LUX hours and 200 Wh/m ^2^ )	97.52	1.00	0.91	98.43	0.026	0.224
Humidity degradation (80% RH at RT for 7 days)	98.45	0.07	0.82	99.27	0.026	0.223
Peroxide degradation (30% H_2_ O_2_ at 80°C for 5 h	89.95	8.57	5.26	95.21	0.027	0.537

#### 3.1.6 Base Degradation Study

Measured and transferred about 50 mg of the sample into a 50 mL volumetric flask, added 5 mL of 5 N NaOH solution, and maintained the sample in a water bath at 80°C for 3 h. The sample was let to cool to room temperature and subsequently neutralized with 5 mL of 5 N HCl solution. Incorporated 30 mL of diluent and subjected the mixture to sonication for 10 min to achieve dissolution. The volume was adjusted to the mark using diluent and well mixed. The sample underwent centrifugation at 5000 rpm for 10 min, after which the supernatant was utilized for HPLC analysis. The resulting chromatogram indicates no substantial deterioration under basic conditions. The outcomes of the percentage test, percentage degradation, mass balance, and peak purity of VEN are presented in Table 4.

#### 3.1.7 Thermal Degradation Study

The samples were maintained in a heated oven at 120°C for 24 h. The exposed samples were weighed and placed, about 50 mg each, into a 50 mL volumetric flask, to which 30 mL of diluent was added, followed by sonication for 10 min to achieve dissolution. The sample was let to equilibrate to room temperature, after which the volume was adjusted to the specified mark using diluent and well mixed. The sample underwent centrifugation at 5000 rpm for 10 min, and the resulting supernatant was utilized for HPLC analysis. The resulting chromatogram indicates no substantial deterioration under heat conditions. The outcomes of the percentage test, percentage degradation, mass balance, and peak purity of VEN are presented in Table 4.

### 3.2 Photodegradation

The sample was subjected to a photostability chamber calibrated at 1.2 million lux hours and 200 Wh/m^2^. The exposed samples were weighed and placed, about 50 mg each, into a 50 mL volumetric flask, to which 30 mL of diluent was added, followed by sonication for 10 min to ensure dissolution. The volume was adjusted to the mark using diluent and well mixed. The sample was centrifuged at 5000 rpm for 10 min, and the supernatant was utilized for HPLC analysis. The resulting chromatogram indicates no substantial deterioration under photolytic conditions. The outcomes of the percentage test, percentage degradation, mass balance, and peak purity of VEN are presented in Table 4.

### 3.3 Degradation Due to Humidity

The specimens were stored in a humidity chamber regulated at 25°C and 80% relative humidity for a duration of 7 days. The exposed samples were weighed and placed, about 50 mg each, into a 50 mL volumetric flask, to which 30 mL of diluent was added, followed by sonication for 10 min to achieve dissolution. The sample was let to cool to room temperature, after which the volume was adjusted to the mark using diluent and well mixed. The sample underwent centrifugation at 5000 rpm for 10 min, and the resulting supernatant was utilized for HPLC analysis. The resulting chromatogram indicates no substantial deterioration in humid circumstances. The outcomes of the percentage test, percentage degradation, mass balance, and peak purity of VEN are presented in Table 4.

### 3.4 Degradation of Peroxide

Approximately 50 mg of the sample was weighed and put into a 50 mL volumetric flask, followed by the addition of 5 mL of 30% H_2_O_2_, and the sample was maintained in a water bath at 80°C for 5 h. The sample was let to cool to ambient temperature. Incorporated 30 mL of diluent and subjected to sonication for 10 min to achieve dissolution. The volume was adjusted to the mark using diluent and well mixed. The sample underwent centrifugation at 5000 rpm for 10 min, and the resulting supernatant was utilized for HPLC analysis. The resultant chromatogram demonstrates considerable deterioration under oxidative conditions. The medication exhibits increased susceptibility to oxidative stress conditions based on the aforementioned results. The outcomes of the percentage test, percentage degradation, mass balance, and peak purity of VEN are presented in Table 4.

### 3.5 LOD and LOQ

The LOD and Limit of Quantitation (LOQ) values for VEN and its organic impurities (VEN Impurity–II and VEN Impurity–III) were estimated using signal‐to‐noise ratios of 3:1 and 10:1, respectively. The anticipated LOD concentration for VEN is 0.002, for VEN Impurity–II is 0.003% w/w, and for VEN Impurity–III is 0.0020% w/w. The anticipated LOQ concentration relative to sample concentration for VEN is 0.0118, for VEN Impurity–II is 0.014% w/w, and for VEN Impurity–III is 0.010% w/w.

A precision investigation was conducted by injecting six distinct VEN and associated impurities, and the %RSD was computed for the area response. The %RSD values for VEN, VEN Impurity–II, and VEN Impurity–III are 3.21, 2.91, and 1.03, respectively, and are within the acceptability standards.

### 3.6 Linearity

The linearity of the approach was assessed by injecting spiked standard solutions of VEN at five (*n* = 5) concentration levels, spanning from the LOQ to 150%. In contrast, VEN Impurity–II and VEN Impurity–III were evaluated at five (*n* = 5) concentration levels, ranging from LOQ to 300%. The calibration curve was derived by plotting the peak areas against the concentrations of VEN, VEN Impurity–II, and VEN Impurity–III. The resulting calibration curve exhibited a correlation coefficient exceeding 0.999 for all three substances, indicating that the method is linear. The results are presented in Table 5.

**TABLE 5:** Method Validation data summary.

Parameters	VEN	VEN Impurity – II	VEN Impurity – III
System suitability *n* = 6			
%RSD, Theoretical plates, Tailing Factor	1.2, 114,107, 1.0	NA	NA
Linearity			
Correlation coefficient	0.9999	1.0000	1.0000
LOD (μg/mL)	0.002	0.003	0.002
LOQ (μg/mL), %RSD	0.0118, 3.21	0.014, 2.91	0.01, 1.03
Accuracy (% of recovery) *n* = 3			
LOQ mean, %RSD	106.0, 4.08	97.6, 4.06	103.9, 2.47
50% mean, %RSD	104.2, 2.41	100.5, 3.66	101.3, 4.35
100% mean, %RSD	104.3, 0.12	102.1, 3.86	102.3, 3.47
150% mean, %RSD	104.1, 0.11	NA	NA
300% mean, %RSD	NA	101.8, 1.12	103.9, 0.46
Repeatability %RSD (*n* = 6)			
System precision, %RSD	0.53	NA	NA
Method precision, %RSD	0.70	0.82	1.16
Intermediate precision, overall %RSD	0.82	3.20	3.57
Solution stability			
In sample at 25°C after 24 h, %Difference	0.97	1.28	0.21
In sample at 25°C after 48 h, %Difference	1.40	2.26	4.48
In sample at 2°C–8°C after 24 h, %Difference	0.37	1.13	0.96
In sample at 2°C–8°C after 48 h, %Difference	0.20	2.16	1.21

Abbreviations: NA: not applicable, RSD: relative standard deviation, LOD: limit of detection, LOQ: limit of quantification.

## 4 Precision and Intermediate Precision

### 4.1 System Precision

The system's accuracy was assessed using six duplicate injections of the VEN standard into the instrument, yielding a %RSD of 0.53. The findings from the system precision research indicated that the system is accurate for quantifying VEN in VEN tablets by HPLC, as presented in Table 5.

### 4.2 Method Precision

The accuracy of the technique was assessed by examining a VEN sample solution spiked with impurities at the 100% specification limit relative to the sample concentration, and the %RSD of the area for each impurity was computed. The findings shown in Table 5 demonstrate that the approach is robust for detecting impurities in VEN.

### 4.3 Intermediate Precision

The method's intermediate accuracy was assessed on various days utilizing distinct equipment. The %RSD of VEN and its impurities was computed. The results are presented in Table 5. And verify the VEN's robustness for identifying impurities.

### 4.4 Accuracy

The method's accuracy was assessed by the usual addition technique. The trial was conducted in triplicate at the limits of quantification (LOQ) and at 50%, 100%, and 150% levels, with percentage recoveries computed accordingly. The %recovery values for each impurity varied from 97.6 to 106.0 for VEN and its impurities, all of which met the acceptability standards. The relative standard deviation values for recoveries of all impurities were below 5.0. The findings are presented in Table 5.

### 4.5 Stability of Solutions

The stability of VEN and its impurities was assessed by keeping the spiked samples in securely sealed volumetric flasks at 25°C and 2°C–8°C for durations of 24 h and 48 h. The percentage difference in the area of spiked samples was computed relative to the freshly generated spiked sample solution. The findings are presented in Table 5. It was determined that VEN and its impurities were stable at 2°C–8°C after 24 h.

### 4.6 Robustness Analysis via Quality by Design

**FIGURE 3:**
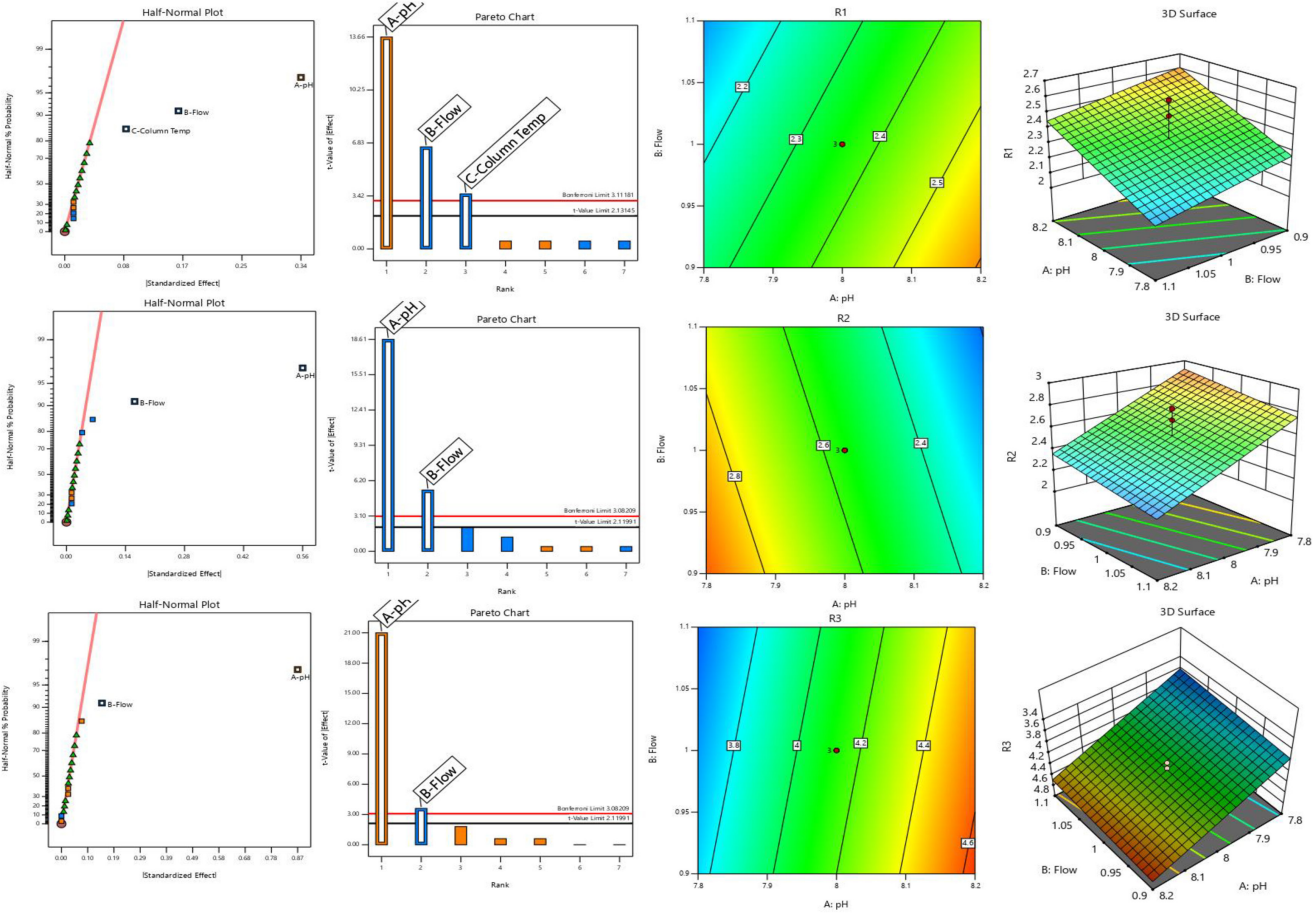
Pareto charts, a half‐normal plot, a 2D contour plot, and a 3D plot for the responses and factors.

The robustness of the developed strategy was confirmed using a statistical analysis grounded in QbD. Critical method parameters (CMPs) were determined via the development of the methodology. The approach employed a buffer pH of 8.0, a column temperature of 30°C, and a flow rate of 1.0 mL/min. The CMPs were alternated between positive and negative modes in compliance with prevailing regulatory standards (USP 621, ICH Q2R2, and EP 2.2.24). The pH of the buffer was adjusted to 7.8, the column temperature was set to 35°C, and the flow rate was changed between 0.90 and 1.10 mL/min. Three sets of resolutions for response variables were selected based on procedure optimization and criticalities: Resolution between impurity C and K (R1), Resolution between VEN impurity II and VEN (R2), and Resolution between VEN and Oxidative VEN (R3). A two‐level randomized complete factorial design was established, incorporating three center points and no blocks. The provided design comprises a total of 19 runs. The runs were executed as scheduled, and the findings were encapsulated in Table 6 and Figure 3.

**TABLE 6:** Quality design tool generated design of experiments runs and responses.

		Factor 1	Factor 2	Factor 3	Response 1	Response 2	Response 3
Std	Run	A:pH	B:Flow	C:Column temp	R1	R2	R3
3	1	8.2	0.9	25	2.7	2.3	4.5
9	2	7.8	0.9	35	2.3	2.9	3.7
7	3	8.2	1.1	25	2.5	2.2	4.4
16	4	8.2	1.1	35	2.4	2.1	4.5
12	5	8.2	0.9	35	2.5	2.4	4.6
10	6	7.8	0.9	35	2.2	2.9	3.8
4	7	8.2	0.9	25	2.6	2.3	4.7
8	8	8.2	1.1	25	2.5	2.3	4.5
17	9	8	1	30	2.5	2.8	4.1
14	10	7.8	1.1	35	2.1	2.8	3.6
18	11	8	1	30	2.6	2.7	4
13	12	7.8	1.1	35	2	2.7	3.5
2	13	7.8	0.9	25	2.3	2.9	3.7
11	14	8.2	0.9	35	2.6	2.4	4.7
19	15	8	1	30	2.6	2.8	4.1
15	16	8.2	1.1	35	2.4	2.2	4.6
6	17	7.8	1.1	25	2.1	2.7	3.7
5	18	7.8	1.1	25	2.2	2.8	3.6
1	19	7.8	0.9	25	2.3	3	3.9

**FIGURE 4:**
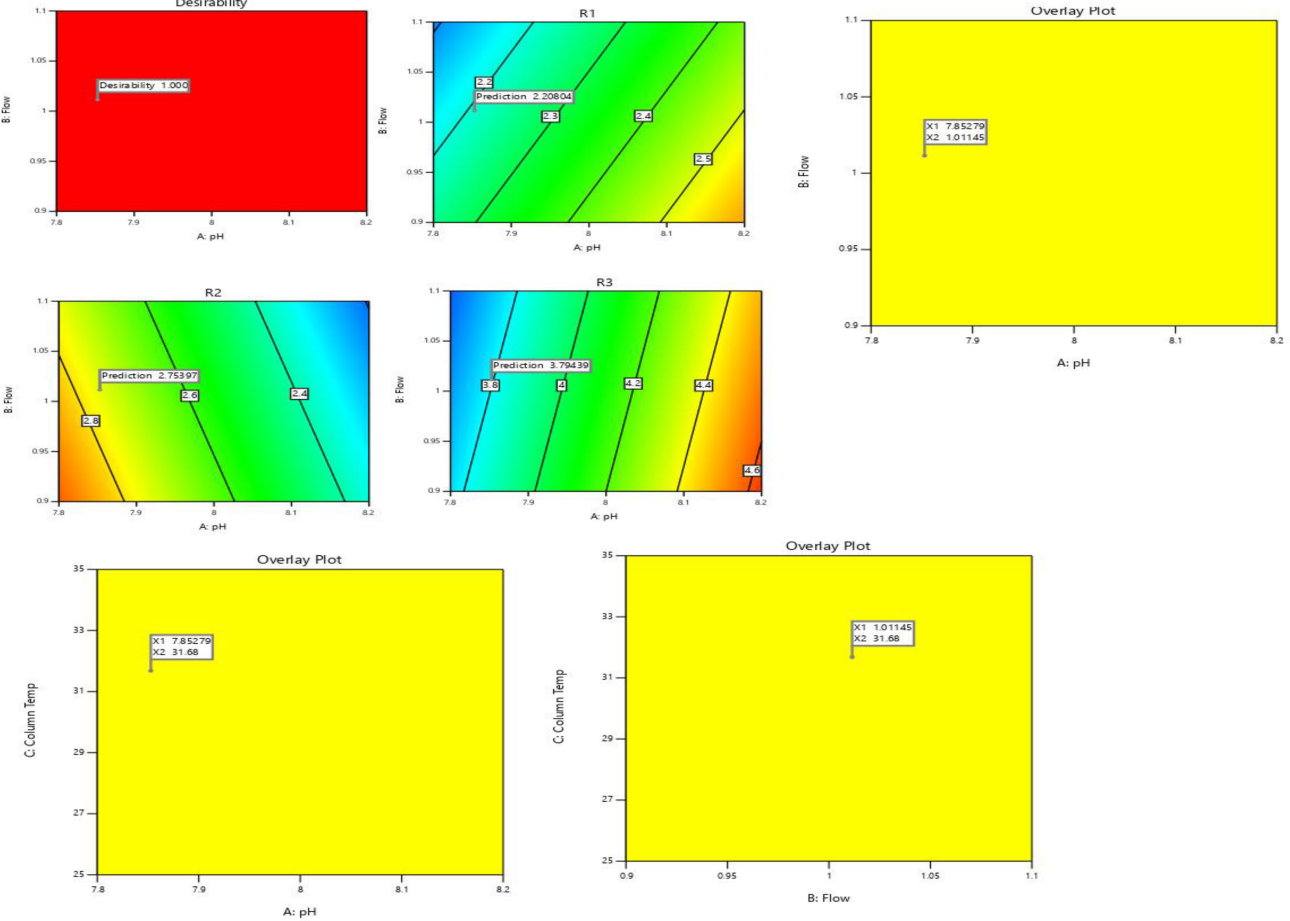
Response surface plots and overlay plots.

The primary impacts were implemented across all replies. The ANOVA Table (Table 7) distinctly indicates that *p*‐values were below 0.05 and statistically significant. The lack of fit was negligible, and all three responses contained substantial data indicative of the model. The difference between expected and actual R2 was found to be less than 0.2. The design space area met the required acceptability criteria in all answers. Figure 3 illustrates Pareto charts, a half‐normal plot, a two‐dimensional contour plot, and a three‐dimensional plot. Figure 4 illustrates reaction surface plots and overlay graphs. R1 determined that the reaction was sensitive to pH, flow, and temperature. The response R2 was determined to be sensitive to pH and flow rate, whereas the response R3 was also shown to be sensitive to pH and flow rate. The findings revealed that all transmuted situations, which did not influence the chromatography and validated technique, were stable.

**TABLE 7:** ANOVA Table.

Response	Source	Sum of squares	df	Mean square	*F*‐value	*p*	
R1	Model	0.5919	3	0.1973	80.84	< 0.0001	Significant
	A‐pH	0.4556	1	0.4556	186.7	< 0.0001	
	B‐Flow	0.1056	1	0.1056	43.28	< 0.0001	
	C‐Column temp	0.0306	1	0.0306	12.55	0.0032	
	Curvature	0.1119	1	0.1119	45.83	< 0.0001	
	Residual	0.0342	14	0.0024			
	Lack of fit	0.0025	4	0.0006	0.1974	0.9341	Not significant
	Pure error	0.0317	10	0.0032			
	Cor total	0.7379	18				
R2	Model	1.37	2	0.6856	187.7	< 0.0001	Significant
	A‐pH	1.27	1	1.27	346.48	< 0.0001	
	B‐Flow	0.1056	1	0.1056	28.92	< 0.0001	
	Curvature	0.1119	1	0.1119	30.62	< 0.0001	
	Residual	0.0548	15	0.0037			
	Lack of fit	0.0231	5	0.0046	1.46	0.2849	Not significant
	Pure error	0.0317	10	0.0032			
	Cor total	1.54	18				
R3	Model	3.15	2	1.58	226.98	< 0.0001	Significant
	A‐pH	3.06	1	3.06	441	< 0.0001	
	B‐Flow	0.09	1	0.09	12.96	0.0026	
	Curvature	0.0086	1	0.0086	1.24	0.2834	
	Residual	0.1042	15	0.0069			
	Lack of fit	0.0275	5	0.0055	0.7174	0.6249	Not significant
	Pure error	0.0767	10	0.0077			
	Cor total	3.27	18				

## 5 Conclusion

The proposed test method benefits the manufacturer of this compound based on the development and validation of experimental data. Pharmaceutical companies can use this method to test their products. This method can separate polar and less polar impurities and has excellent purity. Moreover, the VEN contains nine impurities, all separated without interference. The proposed test method has been validated according to ICH Q2(R2), USP < 1225 > guidelines. We found two potential degradation impurities during the degradation studies. The VEN is stable under acid, base, thermal, humidity, and photolytic conditions. It was sensitive to peroxide conditions. Based on the validation and forced‐degradation data, this method has a stability‐indicating nature. This method is specific, linear, accurate, precise, and robust. It can be used to estimate the VEN‐related substances in the API and finished product and future research workers on the compound.

## Author Contributions

**Ms. Rajeshwari Dandabattina:** methodology, investigation, and writing – original draft. **Dr. Karuna Sree Merugu:** project administration and supervision. **Mr. Lova Gani Raju Bandaru:** investigation, visualisation, and validation. **Dr. Haridasyam SharathBabu:** formal analysis, and data‐curation. **Prof. Rambabu Gundla:** funding acquisition and software. **Dr. Naresh Kumar Katari:** conceptualization, writing – review and editing.

## Ethics Statement

This page does not include any experiments involving human participants or animals conducted by any of the writers.

## Conflicts of Interest

The authors declare no conflicts of interest.
